# Contrasting coloured ventral wings are a visual collision avoidance signal in birds

**DOI:** 10.1098/rspb.2022.0678

**Published:** 2022-07-13

**Authors:** Kaidan Zheng, Dan Liang, Xuwen Wang, Yuqing Han, Michael Griesser, Yang Liu, Pengfei Fan

**Affiliations:** ^1^ School of Life Sciences, Sun Yat-sen University, Guangzhou, People's Republic of China; ^2^ Princeton School of Public and International Affairs, Princeton University, Princeton, NJ 08540, USA; ^3^ Eli Lilly and Company, Indianapolis, IN 46225, USA; ^4^ Department of Biology, University of Konstanz, Konstanz, Germany; ^5^ Centre for the Advanced Study of Collective Behaviour, University of Konstanz, Konstanz, Germany; ^6^ Department of Collective Behavior, Max Planck Institute of Animal Behavior, Konstanz, Germany; ^7^ School of Ecology, Sun Yat-sen University, Shenzhen, People's Republic of China; ^8^ State Key Laboratory of Biological Control, Sun Yat-sen University, Guangzhou, People's Republic of China

**Keywords:** avian flight, signalling, plumage coloration, sensory mechanism, comparative analysis

## Abstract

Collisions between fast-moving objects often cause severe damage, but collision avoidance mechanisms of fast-moving animals remain understudied. Particularly, birds can fly fast and often in large groups, raising the question of how individuals avoid in-flight collisions that are potentially lethal. We tested the collision-avoidance hypothesis, which proposes that conspicuously contrasting ventral wings are visual signals that help birds to avoid collisions. We scored the ventral wing contrasts for a global dataset of 1780 bird species. Phylogenetic comparative analyses showed that larger species had more contrasting ventral wings than smaller species, and that in larger species, colonial breeders had more contrasting ventral wings than non-colonial breeders. Evidently, larger species have lower manoeuvrability than smaller species, and colonial-breeding species frequently encounter con- and heterospecifics, increasing their risk of in-flight collisions. Thus, more contrasting ventral wing patterns in these species are a sensory mechanism that facilitates collision avoidance.

## 1. Background

Collisions between fast-moving objects often cause severe damage or injury, and the heavier these objects are, the more energy is required to avoid an impending collision. In the human world, traffic rules and visual signals (traffic lights, lighting and signalling devices of vehicles) are designed to reduce the risk of traffic collisions. Similarly, group-living animals did evolve mechanisms to reduce the collision risk. For example, group-living bats use echolocation to identify landmarks and flying conspecifics to avoid in-flight collisions [1]. However, how other fast-moving animals such as birds avoid collisions remains understudied.

Birds are well known for their ability to fly, besides a few flightless lineages such as ratites and penguins. Many bird species form large, dense flocks that at times move very fast, for example to escape predators, or make local or long-distance movements. Individuals are therefore at risk of colliding with each other. Indeed, collisions among flying birds can be severe and often fatal, causing injuries that limit foraging or avoiding predators or even death [2,3]. Particularly, large-bodied species and those that form large flocks may be vulnerable to in-flight collisions. These species often collide with man-made obstacles such as power lines [4]. Compared with smaller species, larger species have reduced manoeuvrability due to a higher moment of inertia [5]. Also, larger species fly faster than smaller ones [6,7], reducing the reaction time to avoid collisions. Moreover, species that aggregate in large flocks or breed in colonies frequently encounter con- and heterospecifics, which may increase their collision risk [3,8].

A few studies have assessed how birds avoid in-flight collisions. Budgerigar (*Melopsittacus undulatus*) individuals generally veer to the same direction and keep to a preferred height to avoid collisions [9]. Other species avoid collisions with the help of visual cues, such as European starlings (*Sturnus vulgaris*) or dunlins (*Calidris alpina*) responding to directional changes of the closest flock mates [10,11]. Some migratory birds use flight calls to avoid in-flight collisions when visibility is reduced [12,13]. Notably, white flash marks on the backs and wing coverts of shorebirds, which are typically conspicuous in flight, may signal take-off to flock mates and facilitate coordinated flight movements among flock mates when being attacked by a predator [14]. Thus, birds may also use colour patterns of feathers to avoid in-flight collisions.

**Figure 1.**
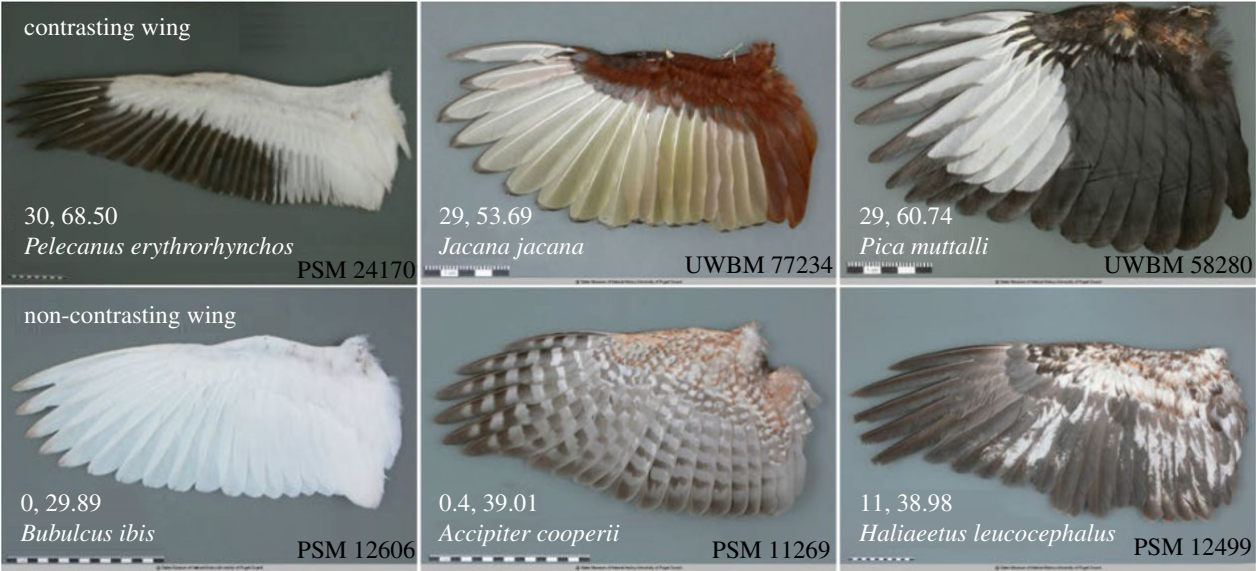
Examples of contrasting and non-contrasting ventral wing patterns. Images are obtained from the Wing & Tail Image Collection at Slater Museum, University of Puget Sound (https://digitalcollections.pugetsound.edu/digital/collection/slaterwing). Numbers on each image are manual score and root-mean-square (RMS) contrast score of each species. (Online version in colour.)

Ventral wing feathers are usually only visible during wing flapping in flight and exhibit a large variation in coloration and patterns among taxonomic groups (figure 1). Interestingly, species in diverse groups exhibit contrasting ventral wings, which might make the flying birds more visible to other individuals underneath, but the function of these patterns remains unexplored. In animals, contrasting coloration increases conspicuousness [15–17], although it can also function as disruptive coloration, increase crypsis or pattern blending [18,19]. In flight, the background of ventral wings is usually the sky, which has a rather uniform coloration that may vary in its brightness. Thus, we predict that contrasting ventral wings could facilitate the visibility of flying individuals to other individuals, particularly those flying underneath or on the same level.

Here, we hypothesize that contrasting ventral wing patterns of birds have evolved as a visual signal that reduces the risk of in-flight collisions. To test this novel collision-avoidance hypothesis, we used a phylogenetically comparative approach to examine whether ventral wing colour contrast is associated with morphological or life-history traits reflecting a higher in-flight collision risk. We predict that (i) larger species have a higher in-flight collision risk than smaller ones, and thus, are more likely to have contrasting ventral wing patterns; also, (ii) species that frequently are in large flocks and/or breed in colonies are predicted to have more contrasting ventral wing patterns than less gregarious species as flocking and colony breeding species are more likely to fly close to a large number of conspecifics than more solitary species. Furthermore, contrasting wing patterns may also have an anti-predator function [20]. Prey can use pursuit deterrence signals [21,22] to inform a predator that it has been detected and thus, an attack likely would fail [23,24]. Thus, we also tested this alternative, non-exclusive hypothesis, which predicts that (iii) species facing high predation pressure have contrasting ventral wings as pursuit-deterrent signals to reduce their predation risk. Finally, as colour patterns are likely more visible at day time than during night, we expect that (iv) diurnal species have higher ventral wing contrast than nocturnal species.

## 2. Material and methods

### (a) Images of avian ventral wings

We collected *n* = 3498 images of ventral wings of *n* = 1980 species (*n* = 1–28 images per species) from three web-based sources, including *n* = 1655 images (*n* = 682 species) of museum specimens (museum ventral wing dataset) from Slater Museum, University of Puget Sound (https://digitalcollections.pugetsound.edu/digital/collection/slaterwing) as well as *n* = 1843 images (*n* = 1298 species) of live birds with natural backgrounds from Wildscreen Arkive (http://www.arkive.org) and the Internet Bird Collection of the Birds of World (https://www.hbw.com/ibc). Images of museum specimens were taken against a standardized background under constant lighting (figure 1), allowing an unbiased quantification of wing colours and patterns [25,26]. The images of live birds were taken under different conditions, preventing the use of objective methods to assess contrast patterns [27].

### (b) Defining manual contrast of ventral wings

We quantified the contrast of ventral wings of all images for each species via manual scoring. We classified the ventral wing patterns into (i) contrasting wings (i.e. wings are composed of two or more distinct colours with each colour distributed in more than 5% of all patches of the ventral wing) or (ii) non-contrasting wings (i.e. wings consist of one colour, or patches without striking contrast; figure 1). Despite these precise definitions, the actual classifications of some images might be ambiguous. To reduce potential subjective bias resulting from a limited number of scorers [14,28–30], and improve the repeatability of the classification, we applied a multi-scorer approach. Thirty volunteering students from the Department of Ecology, School of Life Sciences, Sun Yat-sen University, scored all *n* = 3498 ventral wing images. Images were arranged randomly and species identities were not given during scoring. After a detailed introduction to the definitions of both wing types and the scoring protocol, each student classified all images as contrasting wings or non-contrasting wings. We calculated the manual contrast score of each image as the frequency of being scored as a contrasting wing in all 30 copies of scorings. We then averaged the manual contrast scores of different images for each species.

### (c) Validating manual contrast with root-mean-square contrast in museum ventral wing images

We applied a quantitative method, the root-mean-square (RMS) contrast [31], to validate our manual scoring, using 1655 images from the museum ventral wing dataset. The RMS is calculated as

2.1 $$\mathrm{RMS}={\left[\frac{1}{n-1}\overset{n}{\underset{i=1}{\sum }}{\left(x_{i}-\;\bar{x}\right)}^{2}\right]}^{1/2},$$

where $x_{i}$ represents the grey value of each pixel of the image. RMS contrasts are commonly used to measure the overall contrast of natural images [32], for example to quantify coloration pattern of animals [33]. To do this, we first removed the background of these images and converted them to greyscale, and then calculated the RMS contrast of images. Greyscale converting and RMS calculation were done in the package ‘*cv2*’ in python 3.9. High RMS contrast values indicate high contrast among patches of the ventral wing, while low RMS values indicate a uniform ventral wing pattern.

For the 682 species in the museum ventral wing dataset, the manual contrast score of species was strongly associated with the RMS contrast score (*β* = 0.676 ± s.e. 0.027, *p* < 0.001, *R*^2^ = 0.891 ± s.e. 0.001, averaged from 100 MCMCglmm models each using a randomly selected tree; electronic supplementary material, figure S1), suggesting that our manual contrast score does provide an objective measure of contrast patterns of ventral wings.

### (d) Ecological traits and phylogeny

We collected data on relevant ecological and life-history traits to assess the ecological correlates of ventral wing patterns, including body mass, flock size, coloniality, the number of sympatric avian predators, and activity time of all 1980 species (table 1). We extracted these traits from the Birds of the World [34] (https://birdsoftheworld.org) and other published databases [35,36] (table 1). Body mass (g) was calculated as the mean body mass of both sexes of each species. Flock size was assessed as the average flock size for each species recorded each month in eBird [35]. Coloniality was categorized binary as species that breed in colonial groups versus non-colonial breeders. Predation risk was measured as the number of sympatric avian predators. Previous comparative studies showed that the number of sympatric avian predators predicts global gradients of avian longevity [36,37]. Thus, a larger predator richness probably increased the risk of encountering predators, making the evolution of pursuit-deterrent signals beneficial. Finally, activity time was categorized binary as diurnal versus nocturnal (table 1).

**Table 1.** Predictions for the association between ecological traits and ventral wing patterns.

ecological traits	definition	prediction	data source
body mass (g)	mean body mass of both sexes	larger species are more likely to have higher ventral wing contrast scores than smaller species; larger species have a higher moment of inertia of their wings [5], and thus have a reduced manoeuvrability; moreover, they can fly faster and have upon impact a proportionally higher force [6,7]	[34]
flock size	the average of mean flock size of each month, which is calculated from data on eBird	species that live in large groups have a higher risk of in-flight collisions as they frequently encounter con- and heterospecifics; thus, they are predicted to have higher ventral wing contrast scores than less flocking species	[35]
coloniality	colonial versus non-colonial breeding	species that breed in colonies have a higher risk of in-flight collisions with conspecifics, and therefore may have higher ventral wing contrast scores than non-colonial breeding species.	[34]
no. sympatric avian predators	the number of sympatric avian predators in the range of species	species facing high predation pressure have higher ventral wing contrast scores as pursuit-deterrent signals to reduce predation risks	[36]
activity time	diurnal versus nocturnal	diurnal species have higher ventral wing contrast scores than nocturnal species, because colour patterns are more visible during the day	[34]

We removed 200 species with missing values of trait data, retaining 665 species in the museum ventral wing dataset and 1780 species in the full ventral wing dataset for further analyses [38].

### (e) Statistical analyses

All statistical analyses were performed in R v. 4.0.3 [39]. We used the packages ‘*ggplot2*’ and ‘*ggtree*’ for data visualization [40,41].

**Figure 2.**
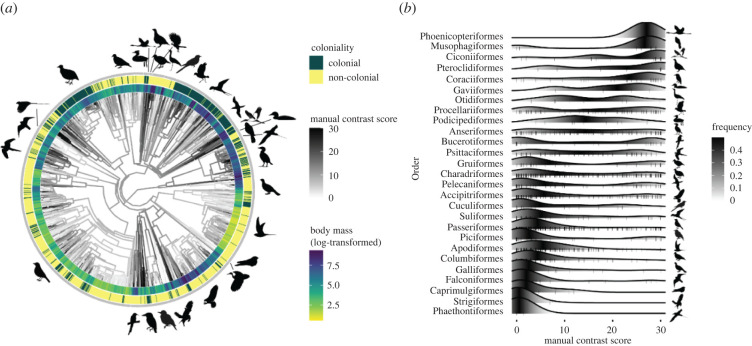
Manual contrast scores of ventral wings and associated ecological traits in birds. (*a*) Distribution of manual contrast scores, log body mass and coloniality across *n* = 1780 species. Colour of branches shows manual contrast scores; darker colour represents higher manual contrast scores. Colour of the inner ring represents log body mass, colour of the outer ring represents coloniality. (*b*) Density distribution of manual contrast score of avian orders. Orders with fewer than two species are excluded (Opisthocomiformes, Coliiformes: *n* = 1; Eurypygiformes: *n* = 2). (Online version in colour.)

To test the collision-avoidance hypothesis and the anti-predation hypothesis, we implemented Bayesian generalized linear mixed models in MCMCglmm [42]. The phylogenetic relatedness among species was included as a random effect in these models. We constructed a model for the full ventral wing dataset (*n* = 1780 species), with manual contrast score as the response variable, and body mass, flock size, coloniality, the number of sympatric avian predators and activity time as predictors. We also included the interaction between coloniality and body mass in the model, with all continuous predictors being centred. Body mass and flock size were log-transformed, while the number of sympatric avian predators was sqrt-transformed to achieve normality. We did not detect co-linearity among the predictors as all variance inflation factors (VIF) were less than 1.5 in all analyses [43]. To account for phylogenetic uncertainty, we ran 100 models using 100 randomly selected phylogenetic trees downloaded from birdtree.org [44]. We used a Gaussian distribution and a parameter expanded prior (*R* = list(*V* = 1, *ν* = 0.002), G = list(G1 = list(*V* = 1, *ν* = 1, *αμ* = 0, *αV* = 1000). The MCMC chains were run for 75 000 iterations, with a 7500 burn-in phase and samples drawn every 40 iterations. Visual inspection of the trace plots revealed model convergence and a low autocorrelation. We averaged the slope coefficient of each predictor and the phylogenetic signal (*λ*) of response variables of the 100 models [45]. The maximum clade credibility tree was generated via the ‘macCladeCred’ function in the R package ‘phangorn’ [46], from 5000 pruned phylogenetic trees of 1780 species downloaded from birdtree.org for visualization (figure 2).

In addition, we ran two models with the same predictors mentioned above for the museum ventral wing dataset (*n* = 665 species), with the manual contrast score and the RMS contrast score as the response variable. RMS contrast was also log-transformed to achieve normality. Both models showed quantitatively the same results (see electronic supplementary material, table S1), confirming that our manual scoring method resulted in unbiased scores.

## 3. Results

### (a) Contrast patterns of ventral wing images

Overall, the mean manual contrast score of all 1980 species was 9.69 ± s.e. 0.23 (range: 0–30). After removing species with missing values of trait data, we retained *n* = 1780 species (*n* = 3230 images) of *n* = 30 avian orders for further analyses, covering 75% of all extant bird orders. The manual contrast score had a strong phylogenetic signal (Pagel's *λ* = 0.77; figure 2*a*). Species with high-contrast ventral wing patterns were mainly found in Phoenicopteriforme, Musophagiformes, Ciconiiformes, Pteroclidiformes and Coraciiformes (figure 2*b*). In contrast, species with low-contrast ventral wing scores were mainly found in Suliformes, Passeriformes, Piciformes, Apodiformes, Columbiformes, Galliformes, Falconiformes, Caprimulgiformes, Strigiformes and Phaethontiformes (figure 2*b*).

### (b) Ecological correlates of ventral wing contrast scores

**Figure 3.**
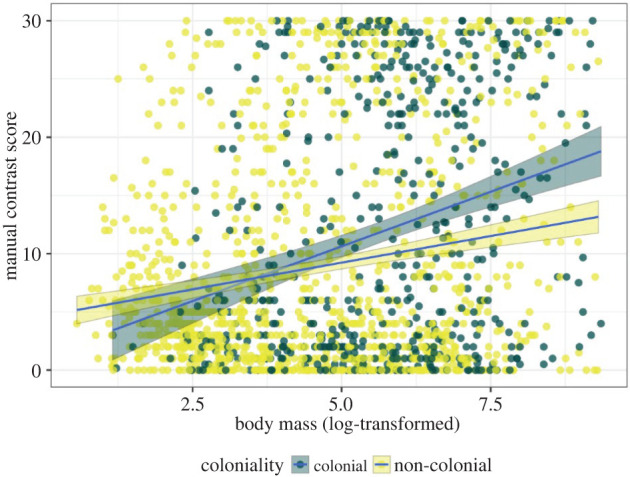
The effect of the interaction between body mass and coloniality on manual contrast scores of ventral wings. *n* = 1780 species. See table 2 for model results. (Online version in colour.)

To test the collision-avoidance and the anti-predation hypotheses, we used phylogenetic mixed models to assess the relationship between manual contrast score and body mass, flock size, coloniality, the number of sympatric avian predators and activity time for 1780 species (table 2; electronic supplementary material, figure S2). These analyses showed that larger species had higher manual contrast scores than smaller species (table 2 and figure 3), as predicted by the collision-avoidance hypothesis. The effect of coloniality interacted with body mass, where particularly large species that breed in colonies had high manual contrast scores (figure 3). In contrast, flock size, the number of sympatric avian predators and activity time were not significantly associated with manual contrast scores. We note that re-running these analyses with the manual and RMS contrast scores in a subset of 648 species with high-quality ventral wings images showed qualitatively the same patterns (see 'Material and methods' and electronic supplementary material, table S1 for details). Also, removing activity time that had an unbalanced sample size between diurnal and nocturnal species did not change the results (electronic supplementary material, table S2).

**Table 2.** Phylogenetically controlled mixed models in MCMCglmm assessing the effect of ecological traits on manual contrast scores of ventral wing patterns in birds (*n* = 1780 species). Model averaging results of 100 Bayesian phylogenetical mixed models, using a set of random trees from http://birdtree.org.

traits	posterior mean	95% CI (lower; upper)	s.e.	*p* MCMC^a^	effective sample size
**intercept**	**10**.**276**	**1.90; 18.58**	**4**.**256**	**0**.**016**	**1688**
activity time (nocturnal versus diurnal^b^)	−7.241	−16.81; 2.39	4.899	0.140	1721
coloniality (colonial versus non-colonial^b^ breeding)	0.789	−0.43; 2.02	0.627	0.210	1687
**log (mass)**	**0**.**700**	**0.07; 1.33**	**0**.**320**	**0**.**029**	**1688**
log (flock size)	0.107	−0.32; 0.54	0.219	0.637	1673
sqrt (no. sympatric avian predators)	0.138	−0.06; 0.34	0.101	0.174	1691
**coloniality: log (mass)**	**0**.**973**	**0.34; 1.61**	**0**.**323**	**0**.**003**	**1704**

^a^*p* MCMC: mean *p*-value of 100 models. Factors with *p* MCMC less than 0.05 are significant and are shown in bold.

^b^Baseline level.

## 4. Discussion

In support of the collision-avoidance hypothesis, phylogenetic mixed models showed that species with a large mass, and among large species particularly those breeding in colonies have high contrasting ventral wings. Contrasting ventral wings may make individuals that are taking off or flying more visible to other individuals, particularly those underneath or on the same level. Thus, contrasting ventral wings could facilitate individuals to accurately assess the speed and direction of individuals flying in their vicinity to reduce the risk of colliding. Notably, this mechanism did evolve independently in multiple avian lineages.

The collision risk is particularly high for large and colonial-breeding species. Heavier species, such as California condor (*Gymnogyps californianus*), great white pelican (*Pelecanus onocrotalus*) and whooping crane (*Grus americana*), have reduced manoeuverability [5,7,47], limiting their ability to make sharp and fast turns to avoid in-flight collisions. These results also suggest that smaller species, such as songbirds, are better at avoiding collision in general, or rely on different mechanisms. Moreover, colonial-breeding species are at times millions of individuals breeding together [48,49]. In these species, large numbers of individuals are moving from and to the same location from different directions at various speeds, making the risk of collision particularly high. Consequently, contrasting ventral wings likely are a beneficial adaptation in colonial breeding species. Furthermore, a number of predators do frequently attack individuals in colonies, for example, African fish eagles (*Haliaeetus voice*) or baboons (*Papio* sp.), regularly try to kill flamingos (Phoenicopteridae). Upon escaping, flamingo individuals display their black-pink contrasting patches on their ventral wings, which could signal the presence of a predator to neighbours, enhance the confusion effect [14] and reduce collisions during escape. Together, the association between contrasting ventral wings in colonial-breeding birds of large body sizes is likely an adaptation to this increased risk of in-flight collisions.

Ventral wing contrasts did not differ between diurnal and nocturnal species, despite that nocturnal species overall have low ventral wing contrasts (range: 0–12; average = 1.54 ± s.e. 0.53). However, only few nocturnal species were included in our analyses, limiting the power to detect a difference. While most nocturnal species breed solitary, sand-coloured nighthawks (*Chordeiles rupestris*) (excluded from the analyses due to the lack of trait data) breed in colonies. This species has highly contrasting ventral wings (manual contrast score = 30), suggesting that contrasting ventral wings may also have a collision avoidance function in nocturnal species, particularly those breeding in colonies.

Interestingly, flock size was not associated with ventral wing contrasts. This finding suggests that species living in large flocks did evolve alternative strategies to avoid in-flight collisions. For example, European starlings (manual contrast score = 1) form flocks of several thousands of individuals, but individuals only respond to the movements of the nearest six to seven individuals [11] and keep sufficient distances to them when the flock is turning and fly at constant speeds [50]. Thus, contrasting ventral wings might not be necessary to avoid collisions for these species. In addition, different selective pressures may prevent smaller species, e.g. songbirds, from evolving contrasting wing patterns. Moreover, flock size data are difficult to sample, and thus, better data are required to further assess its association with ventral wing contrast in the future.

Similarly, the anti-predation hypothesis through pursuit deterrence was not supported by our analyses. The number of sympatric avian predators was not associated with contrasting ventral wing patterns, indicating that these signals do not have a general pursuit deterrence function. This may reflect that birds often use costly signals as pursuit deterrence signals as they honestly signal prey conditions to predators. For example, Eurasian skylarks (*Alauda arvensis*) that sing during encounters with a merlin (*Falco columbiaris*) have an increased chance of successfully evading an attack [51].

The coloration of the upper and ventral wings of birds are exposed to different selection pressures. Compared with ventral wings, upper wings are extensively exposed, and their coloration and patterns have been shown to have predation avoidance and mate choice functions. For example, upper wings of shorebirds such as the buff-breasted sandpiper (*Tryngites subruficollis*) have a cryptic coloration, which might reduce detection by predators. Their ventral wings, however, have a contrasting coloration (manual score = 26). In several species of ducks or red-winged blackbirds (*Agelaius phoeniceus*), males have conspicuously colour patterns on their upper wing, which have been shown to be a sexually selected signal [29,52]. Further work assessing the function of the contrasting upper wings of birds and associated selective pressures could gain more insights into the evolution of contrasting wing colorations.

A potential caveat of our methodology to assess wing contrasting patterns is that many bird species do perceive more colours than humans given their colour vision in tetrachromatic [53–55]. However, most wings with contrasting patterns are composed of black and white patches (for human observers), which similarly results in contrasting patterns in tetrachromatic vision. It is therefore unlikely that our methodology yielded unrepresentative results. Thus, although not taking the tetrachromacy of birds into account, it is reasonable to extend our conclusion to what the birds see.

Finally, we applied a multi-scorer approach, where 30 students scored ventral wing patterns. This number of scorers is larger than previous comparative studies on animal coloration that used a multi-scorer approach [28–30]. Clearly, a large number of scorers reduces a potential scoring bias of a single or a few scorers, and allows using images with natural backgrounds and taken under different light conditions. Thus, a robust definition of colour patterns and high numbers of scorers are recommended for quantifying animal coloration in an objective way when high-quality images are scarce.

## 5. Conclusion

In conclusion, ventral wing contrast patterns of large, colonial-breeding birds have a so-far overlooked signalling function that allows individuals to trace the movements of nearby individuals, thereby reducing the collision risk. Ventral wing coloration likely also has other adaptive functions, including thermoregulation [56,57] and abrasion resistance [58]. Future experimental studies [59] will provide further insights into the mechanisms underlying collision avoidance in gregarious, fast-moving birds and other animals.

## Data Availability

The mean contrasting scores of the ventral wings and ecological data for 1780 bird species and the original code for assessing the relationship between contrasting wing scores and the species' ecological traits for 1780 species, a subset of 1745 diurnal species, and 648 species with high-resolution museum ventral images have been deposited at Zenodo (https://doi.org/10.5281/zenodo.6624690). Electronic supplementary material is available online [60].
